# Acknowledgments

**DOI:** 10.1002/jgf2.221

**Published:** 2018-11-16

It would not be possible to edit the Journal without the advice of many experts. The Editor is grateful to the following people, and members of the Editorial Board, each of whom has reviewed from one to many manuscripts for *Journal of General and Family Medicine* during the past year.

Ariyoshi, Nobuhiro

Azuma, Teruhisa

Endo, Takeshi

Fujikawa, Tatsuya

Fukata, Shuji

Fukuchi, Takahiko

Funakoshi, Hiraku

Furukawa, Naoko

Gomi, Harumi

Gotoh, Jun

Hagiya, Hideharu

Haruta, Junji

Hayashi, Hiroyuki

Hayashi, Tetsuro

Hayashi, Tsuneari

Hirayama, Yoko

Homma, Yoichiro

Honda, Hitoshi

Horibata, Ken

Imaizumi, Kazuyoshi

Inoue, Kazuo

Irioka, Takashi

Ishikawa, Yukiko

Ishimaru, Hiroyasu

Ishimaru, Naoto

Ito, Akihiro

Itoh, Naoya

Iwabuchi, Keisuke

Iwamuro, Masaya

Kajihara, Yusaku

Kakimoto, Keisuke

Kameoka, Junichi

Kanbayashi, Daiki

Kanda, Tatsuo

Kaneko, Makoto

Kaneko, Masahiro

Kanke, Satoshi

Kataoka, Yuki

Kawahara, Hiroaki

Kawano, Fumiaki

Kawashima, Atsushi

Kenzaka, Tsuneaki

Kinjo, Mitsuyo

Kitamura, Yuichi

Kiyota, Masatomo

Kobayashi, Hiroyuki

Kobayashi, Tadashi

Komatsu, Norio

Koyama, Hiroshi

Kutsuna, Satoshi

Manzoor, Kamran

Matsumoto, Masatoshi

Miyamichi, Ryosuke

Miyata, Yasushi

Miyauchi, Akira

MIiyazaki, Kei

Mizuno, Atsushi

Mori, Takahiro

Mutai, Rieko

Nagamatsu, Yasuko

Nakagami, Futoshi

Nakamura, Itaru

Nakamura, Tsukasa

Nakaoke, Ryota

Narita, Masashi

Narumoto, Keiichiro

Nishioka, Hiroaki

Nishisako, Hisashi

Nishizaki, Yuji

Nureki, Shin‐ichi

Oka, Kosuke

Oku, Tomohisa

Otaka, Shunichi

Otsuka, Yuji

Oyama, Yumiko

Ozone, Sachiko

Sada, Ryuichi

Saiki, Takuya

Saito, Tsugumichi

Sakamoto, Yuichi

Saraya, Takeshi

Sasaki, Shugo

Sasaki, Yosuke

Shimizu, Keiki

Shioji, Yasunobu

Shoda, Hirofumi

Sugiyama, Yoshifumi

Tago, Masaki

Takagi, Atsushi

Takahashi, Fumihiko

Takahashi, Jin

Takahashi, Noriyuki

Takamura, Akiteru

Takanashi, Junichi

Takeshima, Taro

Tanabe, Masaki

Tanaka, Taku

Taniguchi, Toshibumi

Tanoue, Hiroki

Tokuda, Koichi

Tomizuka, Taro

Tsuyuguchi, Toshio

Uehara, Yuki

Ui, Mutsuhito

Wada, Mikio

Watanabe, Takamasa

Watanuki, Satoshi

Watari, Takashi

Waza, Kazuhiro

Yabuki, Taku

Yahata, Shinsuke

Yanai, Mitsuru

Yano, Yoshihiko

Yawata, Shinsuke

Yodoshi, Toshifumi

Yokoi, Masayuki

Yoshida, Shuhei

Yoshimi, Yusuke

